# Automatic Polygon Annotation of Plant Objects for Training Dataset Preparation in Green Biomass Segmentation Tasks

**DOI:** 10.3390/jimaging12050192

**Published:** 2026-04-30

**Authors:** Evgeniy Ivliev, Valery Gvindjiliya, Danila Donskoy, Yevgeniy Chayka

**Affiliations:** Laboratory “Modeling and Development of Intelligent Agricultural Engineering Systems”, Don State Technical University, Rostov-on-Don 344000, Russia; vvgvindjiliya@donstu.ru (V.G.); dand22@bk.ru (D.D.); echaika@donstu.ru (Y.C.)

**Keywords:** image segmentation, plant green biomass, YOLO11-seg, automatic annotation, computer vision, agricultural crops

## Abstract

This paper addresses the problem of automated segmentation of plant green biomass in field crop images aimed at improving the accuracy of crop and weed identification. To construct a training dataset for neural network models, an automatic annotation algorithm is proposed, enabling the generation of polygonal object masks without human intervention. The method is based on adaptive analysis of color characteristics of plant fragments with iterative narrowing of the hue range in the HSV color space, combined with an integral quality metric that accounts for the dynamics of contour area and shape. The proposed method achieved an IoU of 93.22% and a DSC of 96.30%, demonstrating a high level of agreement between automatic and manual annotations. The generated masks are used to train segmentation models of the YOLO11-seg family. Models of different scales (n, s, m, l, x) were trained and evaluated using standard metrics, including Intersection over Union (IoU), mAP@0.5, mAP@0.5–0.95, F1-score, and Precision–Recall (PR) curves. Experimental results demonstrate that models trained on automatically generated annotations achieve stable segmentation performance of plant green biomass. The best results were obtained with the YOLO11m-seg model, achieving an F1-score of 0. 772. The results confirm the effectiveness of the proposed approach and demonstrate acceptable segmentation quality, supported by both quantitative metrics and visual analysis. The developed automatic annotation algorithm can be used to expand training datasets in computer vision tasks for agricultural applications.

## 1. Introduction

Modern agriculture is undergoing an active digital transformation, driven by precision farming technologies [1,2] for optimizing agricultural operations, remote monitoring systems for ecosystem control [3,4], and automated data analysis for meta-analysis in agronomy [3,5]. Computer vision and deep learning methods play a key role in these processes [6], enabling the extraction of quantitative characteristics of crop conditions from aerial imagery [7,8,9] and ground-based images [10]. One of the fundamental tasks in agricultural computer vision is the segmentation of green plant biomass, which is essential for evaluating breeding results aimed at increasing biomass yield [11], estimating biomass and vegetative biomass [12,13], and analyzing the spatiotemporal heterogeneity of crops [14].

Despite the significant progress made in neural network segmentation models, their practical application in the agricultural sector is still limited by the quality and size of the training datasets. The preparation of accurate polygon-based annotations for plant objects remains a time-consuming, expensive, and subjective process that requires significant effort and the involvement of domain experts. Errors and inaccuracies in the annotations directly affect the generalization capabilities of the models and lead to a decrease in the quality of segmentation, as has been repeatedly observed in various studies [15,16].

In addition, high-quality polygon-based annotations are crucial not only from a practical point of view, but also to ensure the scientific accuracy, transparency, and reproducibility of computer vision models. Therefore, improving the quality of annotations in the context of reducing the inaccuracy of their polygonal markings is an important factor affecting both the reliability of experimental results and the applicability of the developed models in real agricultural scenarios [17].

Most existing studies focus on the development and comparison of neural network architectures for crop and weed detection and segmentation [18,19,20], while the problem of automating training data generation is often considered secondary. In many cases, manually annotated datasets are used, whereas automatic annotation methods are either based on simple thresholding techniques or are applicable only under controlled conditions (e.g., greenhouse environments), where objects are captured against homogeneous backgrounds without noise from weeds or variable lighting conditions, which are typical for real field scenarios.

Recent studies have also explored the use of deep learning for plant and weed segmentation based on multispectral data. The authors in [21] demonstrated that combining multispectral imaging with PIF-Net and U-Net architectures yields high segmentation accuracy under challenging conditions.

Therefore, the development of methods for automatic or semi-automatic annotation of plant objects, enabling the generation of accurate polygonal masks without human intervention, is a relevant and practically significant task. The use of adaptive algorithms based on the analysis of color and structural characteristics of vegetation can significantly reduce the labor required for dataset preparation.

The aim of this study is to develop and experimentally validate a method for automatic polygonal annotation of plant objects, as well as to evaluate the impact of annotation quality on the performance of neural network models for green biomass segmentation. The results demonstrate that the proposed approach enables efficient generation of training datasets and can be used to expand datasets in agricultural computer vision tasks.

The main scientific contributions of this work are as follows:

1. An automatic algorithm for polygonal annotation of plant objects in field images is developed;
2. An integral quality metric is proposed for selecting the optimal segmentation mask during iterative narrowing of the color range;
3. The performance of YOLO11-seg segmentation models trained on datasets generated using the proposed algorithm is evaluated.

## 2. Materials and Methods

### 2.1. Dataset Description

The LincolnBeet Dataset [22] was used as the initial dataset in this study. It contains color images of early-stage crops (sugar beet) and weeds acquired under field conditions. The images are provided in RGB format and are accompanied by annotations.

The original annotations include bounding box coordinates corresponding to individual plant objects. In this study, these bounding boxes were used as initial information for object localization and subsequent automatic generation of segmentation masks. The dataset does not contain polygonal or pixel-wise annotations.

A preliminary analysis of the training data revealed several systematic inconsistencies and annotation errors. The most common issues include:

- Duplication of bounding boxes for the same object;
- Inaccurate positioning or oversized bounding boxes;
- Incorrect object classification;
- Frequent mislabeling of non-plant objects (e.g., stones and soil artifacts) as weeds.

To address these issues, a data cleaning procedure was applied, including the removal of duplicate annotations, as well as correction or elimination of incorrectly labeled bounding boxes. Table 1 presents statistical information on the number of objects in the original and cleaned training datasets.

**Figure 1:**
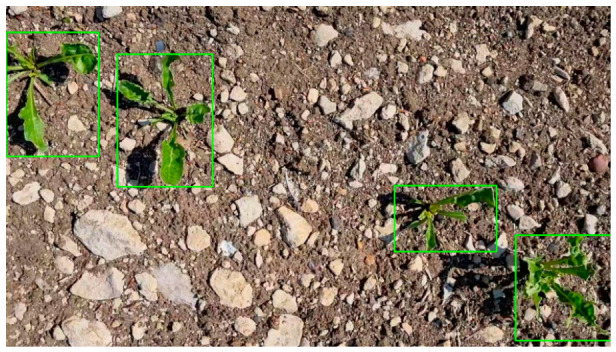
Example of an image with bounding box annotations.

The images are characterized by variability in acquisition conditions, background, and plant growth stages, which necessitates preliminary extraction of regions of interest prior to segmentation. An example of an input image with corrected bounding box annotations is shown in Figure 1.

As can be seen from Figure 1, the images of the training sample are characterized by the non-uniformity of the image background and the presence of shadow artifacts on plant leaves, which created additional natural interference in determining the contour of the object by highlighting channels of color shades.

### 2.2. Preprocessing and Object Extraction

At the preprocessing stage, an XML annotation file in Pascal VOC format is loaded for each image. The coordinates of the bounding boxes are extracted to determine the location of target objects.

**Figure 2:**
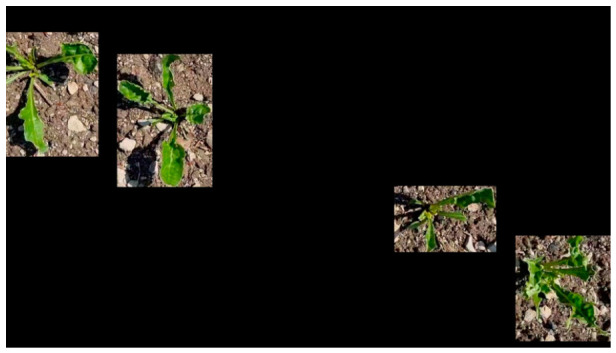
Extraction of target objects.

Each bounding box defines a region of the image containing an individual plant object. These image regions are processed independently and used as input data for the automatic segmentation algorithm. An example of extracted object regions is shown in Figure 2.

### 2.3. Adaptive Color Segmentation in HSV Space

Segmentation of plant objects is performed based on the analysis of color characteristics in the HSV (Hue, Saturation, Value) color space. Converting an image from RGB to HSV allows for more robust separation of vegetation from the background by isolating the hue channel, which is less sensitive to illumination changes.

**Figure 3:**
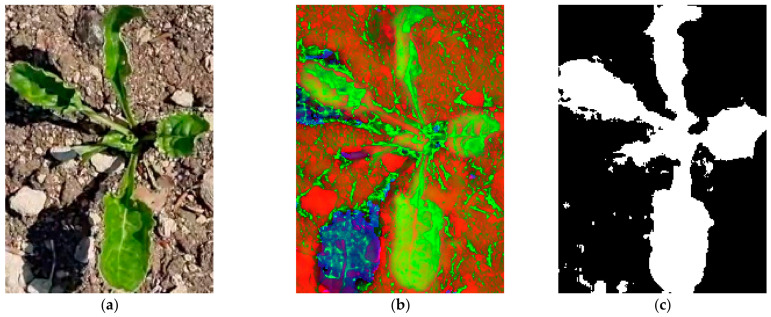
Binary mask generation based on color segmentation: (**a**) original image; (**b**) HSV representation; (**c**) binary mask.

At the first stage, the original image fragment (Figure 3a) is converted into the HSV space (Figure 3b). For each hue range, a binary mask is generated, where pixels falling within the specified range are classified as belonging to the plant object (Figure 3c).

**Figure 4:**
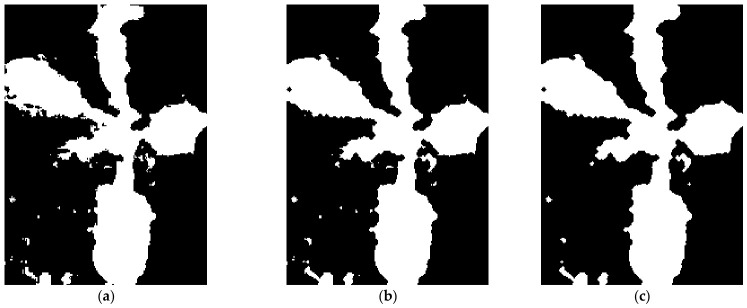
Result of morphological filtering: (**a**) initial mask; (**b**) structure restoration; (**c**) removal of small artifacts.

The obtained binary masks are processed using morphological operations, including *MORPH_OPEN* and *MORPH_CLOSE*, which help remove small noise and restore object connectivity (Figure 4). After morphological filtering, contours are detected and the main connected component corresponding to the plant object is extracted.

**Figure 5:**
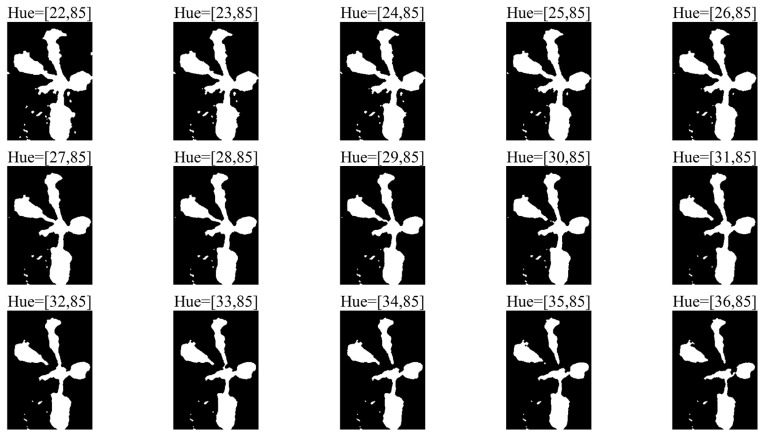
Sequence of intermediate masks generated during hue range narrowing.

The segmentation process is iterative: at each step, the hue range is gradually narrowed. All intermediate masks obtained during this process are stored for further analysis (Figure 5).

### 2.4. Integral Mask Quality Metric

**Figure 6:**
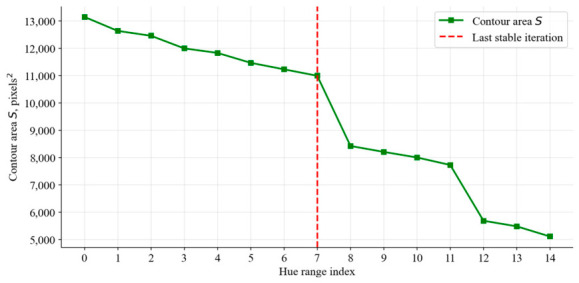
Change in contour area during color range narrowing.

To select the final segmentation mask, we suggest using the integral quality indicator based on an analysis of changes in contour geometry during iterative narrowing of the range of shades. The key parameter here is the coefficient of contour area change between successive iterations

(1) $$k_{area}=\frac{S_{i}}{S_{i-1}}$$

where $S_{i}$ is the contour area at iteration *i*, and $S_{i-1}$ is the contour area at the previous iteration. This coefficient allows for tracking the dynamics of contour area changes and detecting a sharp decrease, which indicates degradation of segmentation quality (Figure 6).

Figure 6 shows that with each iteration of the algorithm for narrowing the color range, the outline of the markup object decreases. At the time of iteration “8”, a large decrease in the contour area of the object is detected, which reflects a partial loss of significant information about the shape of the segmentation object itself. This is the first marker of segmentation degradation and the remaining further changes in contour area with narrowing of the color range.

Additionally, a contour shape metric is computed

(2) $$k_{shape}=\frac{H_{contour}\cdot W_{contour}}{H\cdot W}$$

where H, W are the height and width of the bounding box of the object, and $H_{contour}$, $W_{contour}$—are the height and width of the contour itself.

The final quality score of the mask at iteration *i* is defined as a weighted sum of these metrics:

(3) $$Score=w_{area}\cdot k_{area}+w_{shape}\cdot k_{shape}$$

The coefficient $k_{area}$ reflects the global stability of the segmented region, while $k_{shape}$ characterizes changes in its spatial structure and geometric properties.

**Figure 7:**
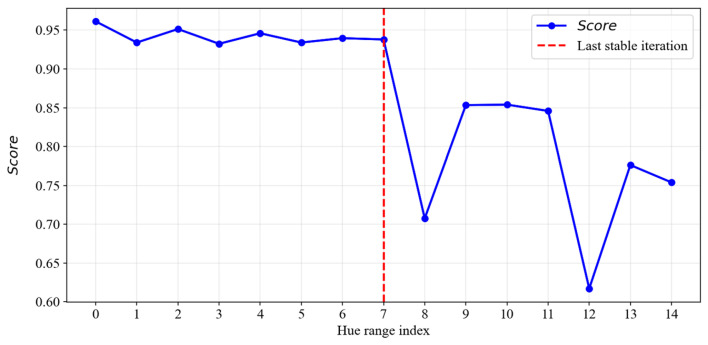
Change in the integral segmentation quality metric during hue range narrowing.

The graph of the integral metric *Score* during the narrowing of the color range is shown in Figure 7. The final mask is selected at the last stable iteration before a sharp drop in the metric value.

**Figure 8:**
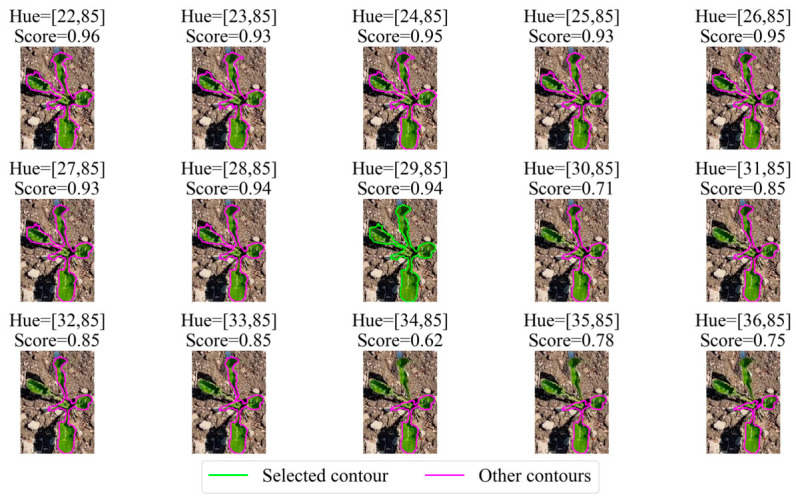
Sequence of intermediate contours formed during hue range narrowing. Image order corresponds to the iteration numbers of the contour quality assessment (from 0 to 14).

The visual representation of contours obtained at each iteration is shown in Figure 8.

As shown in Figure 8, after the seventh iteration of contour refinement, the area of the detected contour changes significantly, and the completeness of the object segmentation contour begins to degrade. A similar decrease in the integral contour quality score is observed for the twelfth image, which is also reflected by the proposed evaluation metrics. The sensitivity of the integral Score to the loss of segmentation data strongly depends on the selected weighting coefficients $w_{area}$ and $w_{shape}$. An analysis of their influence on segmentation quality, as well as the justification for the selected values of $w_{area}$ and $w_{shape}$, is discussed and empirically validated in Section 2.5.

### 2.5. Quantitative Evaluation of Annotation Quality and Selection of Weighting Coefficients

To assess the quality of automatically generated segmentation masks, a quantitative comparison with manually annotated ground truth data was performed. For this purpose, a subset of images was created, for which manual correction of annotations was carried out using the CVAT tool (https://www.cvat.ai/, accessed on 26 April 2026). In total, 581 images were manually annotated. The resulting masks were used as reference data.

The similarity between automatic and manual annotations was evaluated using standard metrics widely applied in image segmentation tasks, including Intersection over Union (IoU) and the Dice Similarity Coefficient (DSC). The IoU metric is defined as the ratio of the intersection area of the automatic mask $A_{Ai}$ and the corresponding manual mask $A_{Mi}$ to the area of their union:

(4) $$IoU=\frac{1}{n}\sum_{i=1}^{n}\frac{\left|{\;A}_{Ai}⋂A_{Mi}\right|}{\left|{\;A}_{Ai}⋃A_{Mi}\right|}\cdot 100\%$$

The DSC characterizes the degree of overlap between two binary masks and is defined as follows:

(5) $$DSC=\frac{1}{n}\sum_{i=1}^{n}\frac{2\left|{\;A}_{Ai}⋂A_{Mi}\right|}{\left|{\;A}_{Ai}\right|+\left|{\;A}_{Mi}\right|}\cdot 100\%$$

The use of these metrics enables an objective evaluation of the proposed automatic annotation method and allows for determining its suitability for generating training datasets in image segmentation tasks. To determine the optimal combination of the weighting coefficients $w_{area}$ and $w_{shape}$, an analysis was conducted in which the coefficient values were varied, and the resulting segmentation quality was evaluated using IoU and DSC metrics (Table 2).

Based on the comparison with manually annotated data, it was determined that the best segmentation performance is achieved with weighting coefficients $w_{area}$ = 0.7 and $w_{shape}$ = 0.3, corresponding to the maximum values of the IoU and DSC metrics.

The obtained results confirm that the contour area variation coefficient $k_{area}$ is the most informative indicator of segmentation quality, as it is highly sensitive to the loss of significant parts of the object. At the same time, the shape coefficient $k_{shape}$ provides an additional contribution by accounting for geometric and structural changes in the segmented object.

In particular, plant objects often exhibit elongated shapes (e.g., leaves). When the hue range is narrowed, such structures may partially disappear, while the total contour area changes only slightly. However, the dimensions of the bounding box may change significantly. In such cases, the coefficient $k_{shape}$ captures variations in spatial structure and enables the detection of partial loss of elongated elements.

Thus, the selected ratio of coefficients ensures a balance between preserving the area stability of the segmented region and maintaining its geometric structure.

### 2.6. Contour Approximation

**Figure 9:**
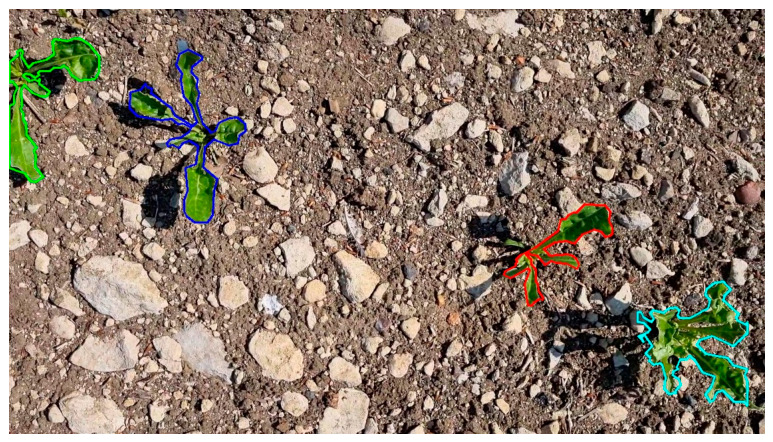
Result of mapping the optimal contour to the original image coordinate space.

The contour corresponding to the selected segmentation mask is mapped back to the coordinate space of the original image, taking into account the position of the bounding box (Figure 9).

To reduce the number of vertices and eliminate excessive detail, the Douglas–Peucker approximation algorithm [23] is applied. This approximation preserves the object shape while reducing the number of points describing the contour.

The resulting contour is converted into a closed polygon and stored as a set of vertex coordinates used to generate segmentation annotations in YOLO format.

### 2.7. Algorithm Formalization and Implementation Parameters

To ensure the reproducibility of the proposed segmentation method, it is presented in the form of a formalized algorithm in which all key processing stages are explicitly defined. The algorithm generates polygonal annotations based on regions specified by bounding boxes using adaptive HSV filtering and contour analysis. The overall procedure for polygon annotation generation is described in Algorithm 1.
**Algorithm 1.** Polygon Annotation GenerationInput: image I, bounding box B Output: polygon P 1: I_crop = crop(I, B) 2: I_hsv = convert_to_HSV(I_crop) 3: H_ranges = {(22 + i, 85)}, i = 0, …, 15 4: best_score = −1 5: history = [] 6: for each (H_low, H_high) in H_ranges do 7:   mask = threshold(I_hsv, H_low, H_high, S = [20, 255], V = [20, 240]) 8:   mask = morphology_close(mask, kernel = 3 × 3, iter = 2) 9:   mask = morphology_open(mask, kernel = 3 × 3, iter = 1) 10:    contours = find_contours(mask) 11:    if contours is empty then continue 12:    C = max_area_contour(contours) 13:    area = contour_area(C) 14:    if area < 50 then continue 15:    bbox = bounding_rect(C) 16:    k_shape = (bbox.w * bbox.h)/(I_crop.w * I_crop.h) 17:    if first iteration then 18:     k_area = 1 19:    else 20:     k_area = area/prev_area 21:    score = 0.7 * k_area + 0.3 * k_shape 22:    prev_area = area 23:    store (mask, score) in history 24: best_idx = find_first_significant_drop(history, 0.2) 25: best_mask = history[best_idx].mask 26: C_best = max_contour(best_mask) 27: epsilon = 0.0015 * perimeter(C_best) 28: P = approx_polygon(C_best, epsilon) 29: return P

The algorithm includes a set of tunable parameters that control the stages of color filtering, morphological processing, and contour approximation. The following parameter settings were used in this study:

- HSV hue range: H ∈ [22, 85] with a step of 1 (14 iterations);
- Saturation and value ranges: S ∈ [20, 255], V ∈ [20, 240];
- Morphological processing:
  Closing (kernel 3 × 3, 2 iterations);
  Opening (kernel 3 × 3, 1 iteration).
- Minimum contour area: 50 pixels;
- Scoring function: Score = 0.7·$k_{area}$ + 0.3·$k_{shape}$;
- Selection criterion: first significant drop in Score (20% of score range);
- Douglas–Peucker approximation parameter: ε = 0.0015·perimeter.

### 2.8. Neural Network Architecture and Training Parameters

To solve the problem of green biomass segmentation, the YOLO11-seg neural network architecture [24,25] was used, designed for single-stage object detection and segmentation on raster images.

The YOLO11-seg model employs an improved feature extraction block that generates multi-scale feature maps while reducing the number of parameters compared to previous versions. This enables efficient processing of images with high variability in the size and shape of plant objects. Additional feature aggregation modules enhance the model’s robustness to heterogeneous backgrounds and partial object occlusions, which are typical for field crop images.

In the output part of the model, optimized convolutional operations are applied to reduce computational load while maintaining the accuracy of predicting object coordinates, classes, and segmentation masks. Altogether, these architectural solutions provide a balance between inference speed and segmentation quality.

Within this study, several configurations of the YOLO11-seg model (n, s, m, l, x) were considered, differing in the number of trainable parameters and computational complexity. The main characteristics of the models, including the number of parameters and computational cost in terms of FLOPS, are presented in Table 3.

Training of the neural network models was performed on a workstation equipped with an NVIDIA RTX 3050 GPU (NVIDIA Corporation, Santa Clara, CA, USA) with 8 GB of VRAM, an Intel Core i7-13700F CPU (Intel Corporation, Santa Clara, CA, USA), and 32 GB of DDR5 RAM (KingSpec, Shenzhen, China). The PyTorch framework (version 2.9.1) and the Ultralytics YOLO library (version 8.4.8) were used as the software environment. Training was conducted under the Ubuntu 22.04 operating system with CUDA support (version 13.0).

The dataset was divided into training, validation, and test subsets consisting of 2713, 581, and 582 images, respectively. All images have a spatial resolution of 1137 × 640 pixels. To ensure reliable evaluation, the validation subset was manually annotated. The batch size was selected based on GPU memory limitations and set to 8. Training was performed for 300 epochs using the stochastic gradient descent (SGD) optimizer with a momentum of 0.937 and a weight decay of 0.0005.

## 3. Results and Discussion

### 3.1. Annotation Quality Evaluation

To evaluate the effectiveness of the proposed method, a comparison with several classical approaches was conducted (Table 4), including:

- HSV-based threshold segmentation;
- Otsu global binarization;
- Adaptive thresholding;
- K-Means clustering.

The obtained results demonstrate a significant advantage of the proposed method over classical approaches. The closest performance is achieved by HSV-based thresholding with fixed parameters; however, it underperforms the proposed method by more than 16% in terms of IoU.

### 3.2. Evaluation of the Quality of Neural Network Training

To evaluate the performance of the trained YOLO11-seg models, a set of standard segmentation metrics was used, including Intersection over Union (IoU), mAP@0.5, mAP@0.5–0.95, the F1-score, and Precision–Recall (PR) curves. These metrics allow for an objective assessment of both object localization accuracy and the quality of segmentation mask reconstruction compared to the ground truth annotations.

**Figure 10:**
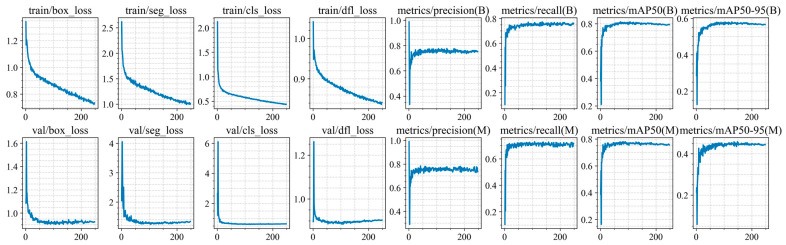
Training and validation results of the YOLO11n-seg model.

**Figure 11:**
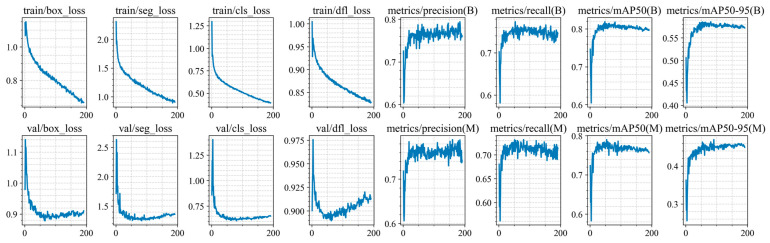
Training and validation results of the YOLO11s-seg model.

**Figure 12:**
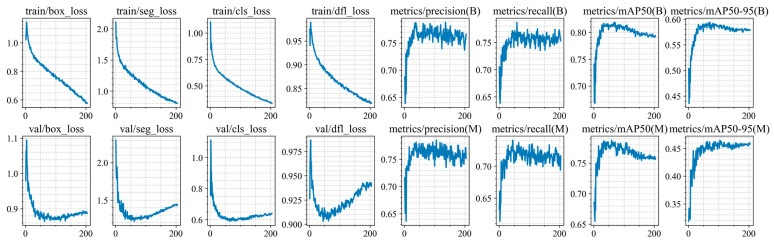
Training and validation results of the YOLO11m-seg model.

**Figure 13:**
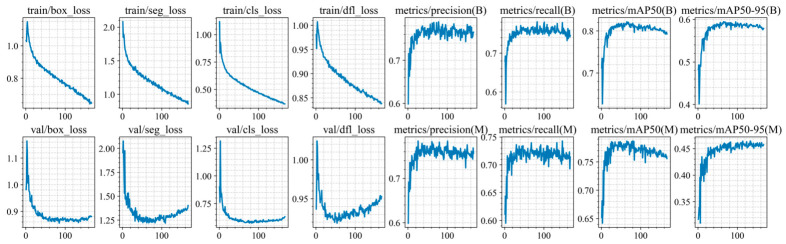
Training and validation results of the YOLO11l-seg model.

**Figure 14:**
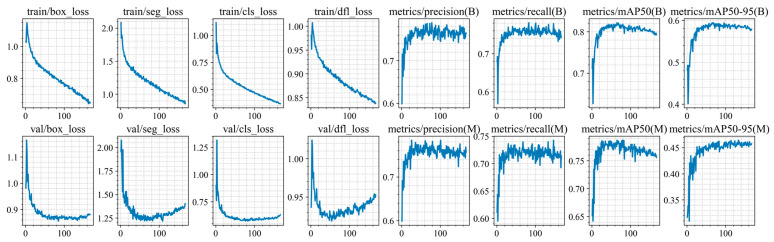
Training and validation results of the YOLO11x-seg model.

Training of the YOLO11n-seg, YOLO11s-seg, YOLO11m-seg, and YOLO11l-seg models was conducted for up to 300 epochs, but was stopped early due to the lack of improvement in segmentation and classification metrics on the validation set (at epochs 248, 191, 204, 167, and 171 for each architecture, respectively). The training and validation results are shown in Figure 10, Figure 11, Figure 12, Figure 13 and Figure 14.

At the initial stages of training, a rapid increase in mAP@0.5 values is observed, indicating fast adaptation of the models to the data structure. Subsequently, the metric curves reach a plateau (in the range of 70–250 epochs), demonstrating convergence to a stable state without pronounced signs of overfitting. However, at later stages of training, a sharp decline in this metric is observed. Based on this trend, an early stopping criterion was applied, and the model with the best recorded performance was selected for further use.

For all models, the best performance in terms of mAP@0.5 is achieved at intermediate training stages (between epochs 47 and 72), with peak values ranging from 0.814 to 0.822. However, further training leads to a noticeable decline in accuracy, with final mAP@0.5 values decreasing by 0.02–0.029. This behavior clearly indicates the presence of overfitting.

At the same time, the analysis of the Distribution Focal Loss (val/dfl_loss) shows a slight increase after reaching its minimum value. Although the magnitude of this increase is relatively small (Δ = 0.01–0.035), it consistently correlates with the degradation of mAP across all models.

It is important to note that the increase in val/dfl_loss is relatively minor compared to the drop in mAP. This indicates that overfitting in this case is not severe and affects overall detection quality more than localization stability.

**Figure 15:**
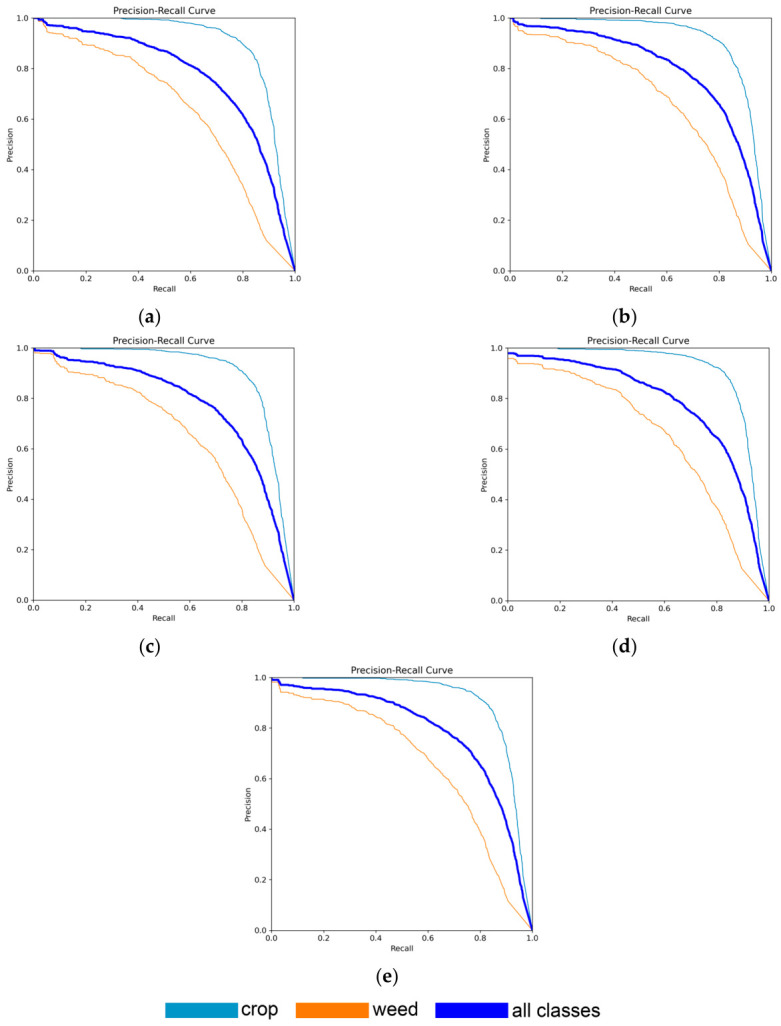
Precision–Recall curves of neural network models: (**a**) YOLO11n-seg: crop 0.901; weed 0.637; all classes 0.769; (**b**) YOLO11s-seg: crop 0.906; weed 0.666; all classes 0.786; (**c**) YOLO11m-seg: crop 0.906; weed 0.647; all classes 0.776; (**d**) YOLO11l-seg: crop 0.911; weed 0.650; all classes 0.780; (**e**) YOLO11x-seg: crop 0.910; weed 0.662; all classes 0.786.

An important role in evaluating model performance was played by PR curves (Figure 15). This metric is widely used in object detection and segmentation tasks and allows for analyzing the trade-off between precision and recall at different confidence thresholds. The area under the PR curve corresponds to the Average Precision (AP) for a given class, while averaging AP over all classes at a fixed IoU threshold yields the mAP@0.5 metric. PR curves provide a more comprehensive assessment of model performance compared to traditional accuracy, especially in the presence of imbalanced data and objects of varying scales.

Averaging AP across all classes at a given IoU threshold forms the mAP@0.5 metric. The highest AP values were achieved by the YOLO11s-seg and the YOLO11x-seg models. PR curves enable a more complete evaluation of model performance compared to conventional accuracy, particularly under class imbalance and varying object sizes.

**Figure 16:**
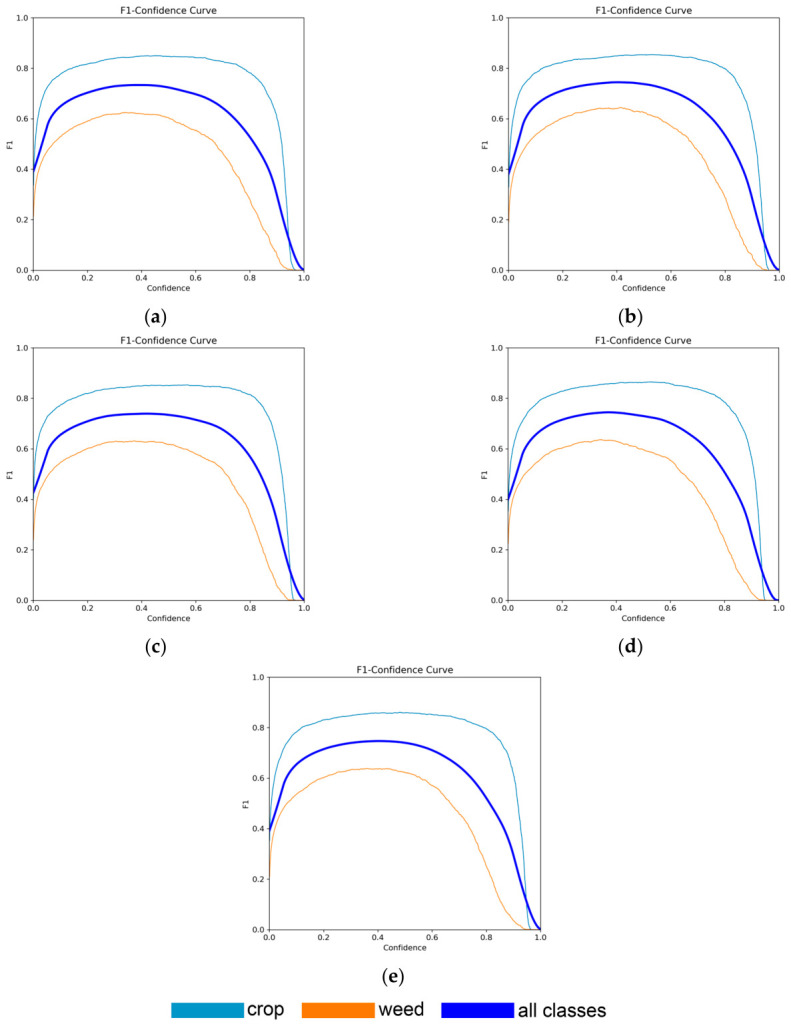
F1–Confidence curves of neural network models: (**a**) YOLO11n-seg: crop 0.91; weed 0.63; all classes 0.76; (**b**) YOLO11s-seg: crop 0.91; weed 0.63; all classes 0.76; (**c**) YOLO11m-seg: crop 0.91; weed 0.63; all classes 0.76; (**d**) YOLO11l-seg: crop 0.91; weed 0.63; all classes 0.76; (**e**) YOLO11x-seg: crop 0.91; weed 0.63; all classes 0.76.

Additionally, the F1-score was calculated to assess the balance between precision and recall, which is especially important in the presence of uneven class distributions. The best F1-score (0.75) was achieved by the YOLO11x-seg model at a confidence threshold of 0.404 (Figure 16).

A quantitative comparison of YOLO11 segmentation models is presented in Table 5. The evaluation was performed on the validation dataset using standard metrics, including Precision, Recall, mAP@0.5, mAP@0.5:0.95, and F1-score.

The results indicate that all considered model variants demonstrate consistently high performance, with mAP@0.5 values exceeding 0.814. The best mAP@0.5 score (0.822) was achieved by the YOLO11l-seg and YOLO11x-seg models, while the highest F1-score (0.772) was obtained by the YOLO11m-seg model. At the same time, the differences between the models are relatively small, suggesting that increasing model complexity does not lead to a significant improvement in segmentation performance for the considered task.

**Figure 17:**
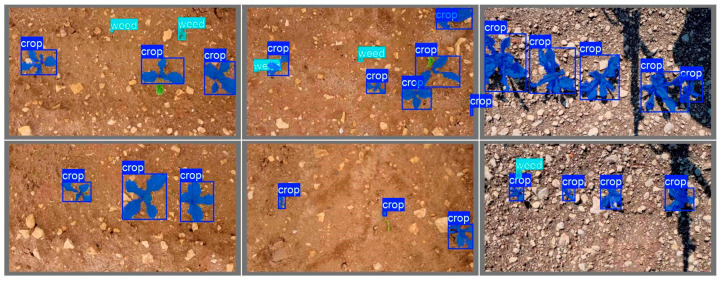
Visualization of annotations for weeds and cultivated plants.

**Figure 18:**
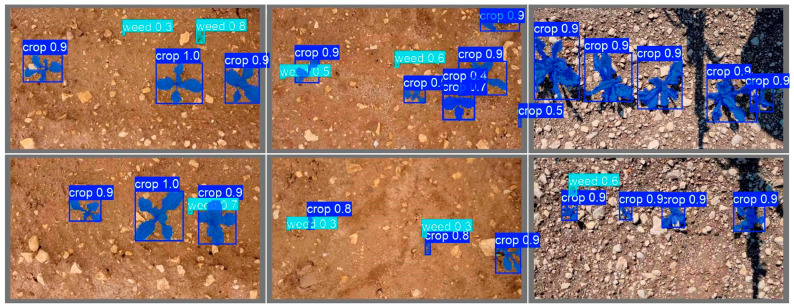
Segmentation and classification results of weeds and plants using the YOLO11x-seg model.

Additional visual evaluation of segmentation and classification quality was performed on a set of test images. Figure 17 shows the visualization of the original annotations of weeds and cultivated plants used during training. Figure 18 presents the results of segmentation and classification produced by the YOLO11x-seg model.

**Figure 19:**
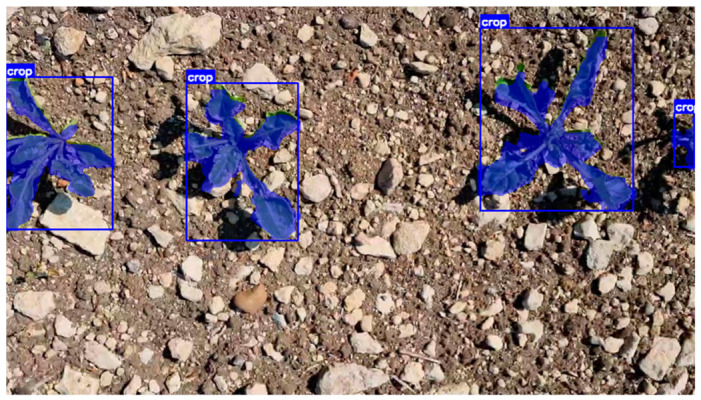
Segmentation and classification results of weeds and plants on higher-resolution images using the YOLO11x-seg model.

The results of segmentation and classification on higher-resolution images using the YOLO11x-seg model are shown in Figure 19.

Thus, within this section, a neural network model for green biomass segmentation based on the YOLO11-seg family was developed and trained.

## 4. Conclusions

This paper proposes a method for automatic polygon labeling of plant objects in images of field crops, which is aimed at reducing the complexity of preparing training datasets for green biomass segmentation tasks. The method is based on adaptive color segmentation in the HSV space, with a sequential narrowing of the hue range, and the selection of an optimal mask based on an integral metric that takes into account changes in the area and shape of the contour.

It is shown that the developed algorithm allows for the creation of correct polygon masks without the need for operator intervention, which can be used for training neural network segmentation models. An experimental evaluation conducted on YOLO11-seg models of various configurations demonstrated that the automatically generated labels provide stable training quality. The best results were achieved for the YOLO11x-seg model, with an F1-measure of 0.75, which confirms the practical applicability of the proposed approach.

The obtained results indicate the potential for using the developed method to expand and update training datasets in agricultural computer vision tasks, even with limited manual labeling. Automatic polygon labeling can be particularly useful when working with large image datasets and adapting models to new imaging conditions.

The proposed method demonstrates satisfactory performance in accomplishing its intended task. However, it is based on color segmentation in the HSV color space, which makes it sensitive to illumination conditions, shadows, and background variability. For instance, bright soil artifacts (e.g., stones) or dark shadows may lead to incorrect segmentation results. In such cases, adaptation based solely on a single hue channel is insufficient; therefore, future research will focus on incorporating adaptive processing of the saturation and value channels to improve robustness under varying environmental conditions.

## Figures and Tables

**Table 1:** Static information about datasets.

Dataset Version	Images	Crops	Weeds
Original dataset	3876	14,403	19,692
Cleaned dataset	3876	13,547	18,877

**Table 2:** Effect of weighting coefficients on segmentation quality.

$w_{area}$	$w_{shape}$	IoU, %	DSC, %
1.0	0.0	90.19	94.61
0.9	0.1	91.22	95.18
0.8	0.2	92.01	95.63
0.7	0.3	93.22	96.30
0.6	0.4	92.41	95.86
0.5	0.5	91.35	95.27
0.4	0.6	90.10	94.54
0.3	0.7	89.05	93.93
0.2	0.8	88.12	93.36
0.1	0.9	87.28	92.87
0.0	1.0	84.23	91.08

**Table 3:** Performance metrics of YOLO11-seg neural network models.

Model	FLOPS (×10^9^)	Parameters (×10^6^)
YOLO11n-seg	9.7	2.9
YOLO11s-seg	33.0	10.1
YOLO11m-seg	113.2	22.4
YOLO11l-seg	132.2	27.6
YOLO11x-seg	296.4	62.1

**Table 4:** Comparison of segmentation methods.

Method	IoU, %	DSC
The proposed method	93.22	96.30
HSV thresholding	76.42	85.53
K-Means	47.84	63.29
Adaptive threshold	43.49	59.64
Otsu binarization	39.76	55.22

**Table 5:** Quantitative comparison of YOLO11 segmentation models on the validation dataset.

Model	Precision	Recall	mAP@0.5	mAP@0.5:0.95	F1-Score
YOLO11n-seg	0.774	0.755	0.814	0.579	0.764
YOLO11s-seg	0.787	0.735	0.819	0.583	0.760
YOLO11m-seg	0.783	0.761	0.819	0.592	0.772
YOLO11l-seg	0.770	0.760	0.822	0.594	0.765
YOLO11x-seg	0.764	0.755	0.822	0.596	0.759

## Data Availability

The generated dataset with polygonal segmentation annotations of crop and weed plants, produced using the proposed algorithm and based on the LincolnBeet Dataset, is publicly available on Hugging Face at: https://huggingface.co/datasets/ivliev123/polygonal_marking_plant_objects (accessed on 2 February 2026). These resources include the automatically generated polygonal masks, visualization results, and supporting materials necessary to reproduce the experiments and validate the results reported in this study.
